# *Malassezia* Fungi Are Specialized to Live on Skin and Associated with Dandruff, Eczema, and Other Skin Diseases

**DOI:** 10.1371/journal.ppat.1002701

**Published:** 2012-06-21

**Authors:** Charles W. Saunders, Annika Scheynius, Joseph Heitman

**Affiliations:** 1 Procter and Gamble Beauty Science, Miami Valley Innovation Center, Cincinnati, Ohio, United States of America; 2 Translational Immunology Unit, Department of Medicine Solna, Karolinska Institutet, Stockholm, Sweden; 3 Duke University Medical Center, Durham, North Carolina, United States of America; The University of North Carolina at Chapel Hill, United States of America

## Introduction

**Figure 1:**
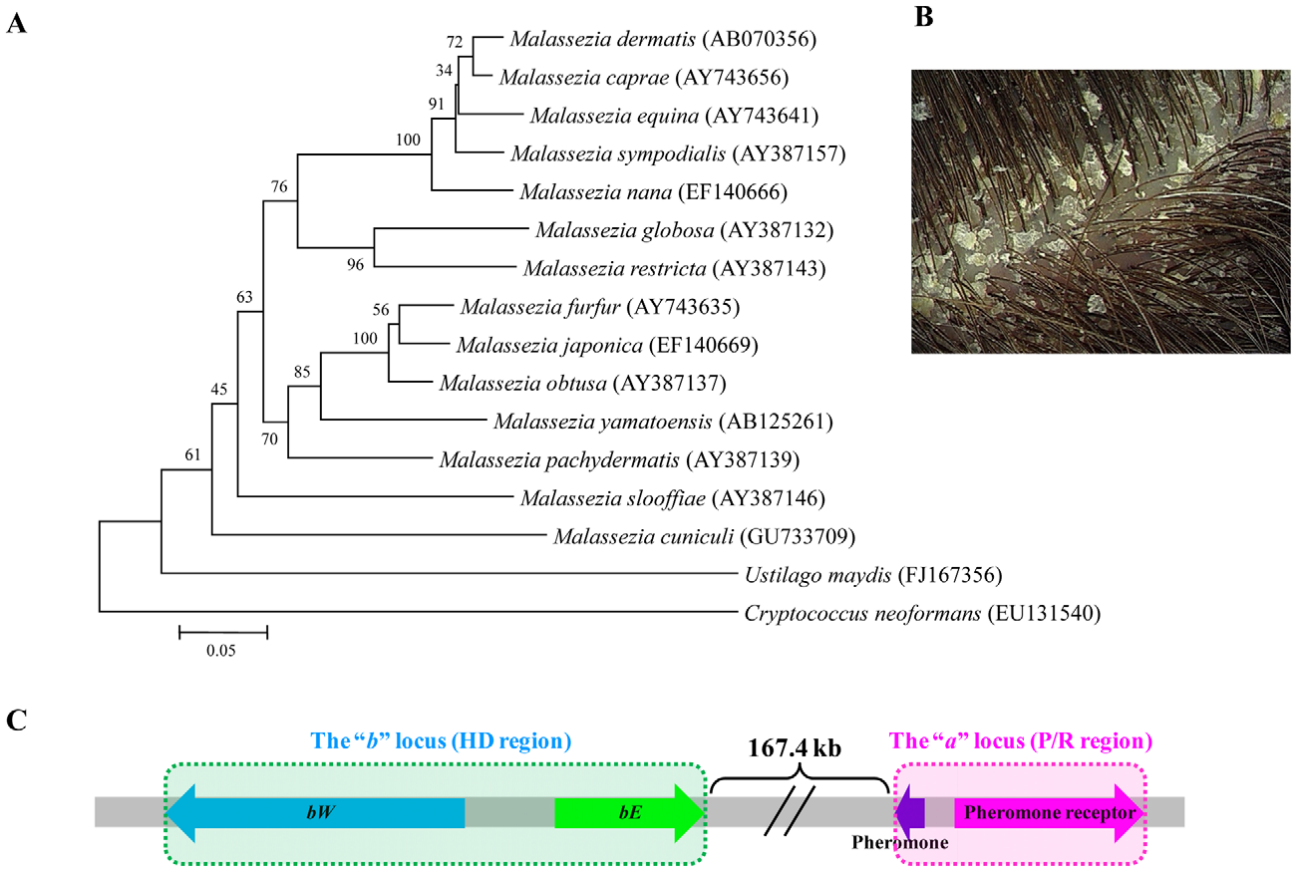
*Malassezia* phylogeny, impact on human skin, and mating type (*MAT*) locus. (A) An ITS sequence-based phylogenetic tree of 14 *Malassezia* species inferred using neighbor joining method and a 500 replicate bootstrap analysis. Closely related *Ustilago maydis* and *Cryptococcus neoformans* were used as outgroups. (B) *Malassezia*-associated dandruff (photograph by Holly Krigbaum). (C) *M. globosa MAT* locus, comprising the *bW*- and *bE*-encoded transcription factors (the *b* locus) and genes for a pheromone and pheromone receptor (the *a* locus).

*Malassezia* is a monophyletic genus of fungi found on the skin of 7 billion humans and associated with a variety of conditions, including dandruff, atopic eczema (AE)/dermatitis, pityriasis versicolor, seborrheic dermatitis, and folliculitis ([1], [2]; Figure 1). In immunocompromised hosts *Malassezia* can also cause systemic infections. There are 14 currently recognized species of *Malassezia*, eight of which have been associated with humans, four of these commonly [3]. *Malassezia* spp. are Basidiomycetous fungi, as are most species of fungi readily seen on a walk through the forest. Among the Basidiomycota, only *Malassezia* and *Cryptococcus* are frequent human pathogens. However, their adaptations to humans are presumed to be independent: *Malassezia's* closest relatives are plant pathogens: the smuts and rusts, whereas the closest relatives for *Cryptococcus* pathogenic species are fungal saprotrophs associated with trees and insects. We summarize here a cellular and molecular description of some interactions of *Malassezia* with humans and speculate on properties (release of allergen-containing nanovesicles, mating) that may be critical to *Malassezia* virulence.

## What Is Known about the Gene Content of *Malassezia*?

A genome sequence of *Malassezia globosa* reveals as small a genome size as any free-living fungus, with only 4,285 genes and spanning just ∼9 Mb [4]. This small genome size may reflect adaptation to the organisms' limited niche, the skin of warm-blooded vertebrates [5]. While many of the genes for biosynthetic enzymes are present, *M. globosa* is the only free-living fungus known to lack a fatty acid synthase gene [4]. With a plethora of lipase genes, *M. globosa* likely satisfies its lipid requirement by hydrolysis of sebum triglycerides. Within the genus, only *Malassezia pachydermatis*, isolated from dogs and other non-human animals [5], is known to grow in the absence of exogenous lipid [1]. It will be interesting to learn whether this atypical species contains a fatty acid synthase gene similar to that found in the close relative *Ustilago maydis* and whether the habitat requirements of *M. pachydermatis* are, as a consequence, less stringent by relieving the requirement for exogenous lipids. While it is possible to culture *Malassezia* species axenically under laboratory conditions by providing exogenous lipids that mimic those available on human skin, some species are still quite fastidious, suggesting in vitro culture conditions may not be optimized.

## Are *Malassezia* Species Related to Dermatophytes or Other Fungi Living on Vertebrate Skin?

Strictly no, despite the similarity of habitat. Dermatophytes such as *Trichophyton rubrum*, the cause of athlete's foot infections, colonize and infect the skin and nails. The dermatophytes are ascomycetous fungi that are related phylogenetically to the dimorphic fungal pathogens. By contrast, the *Malassezia* species are superficial commensals of the skin but can provoke inflammatory reactions resulting in symptomatic skin diseases (folliculitis, dandruff, eczema) in humans and other animals. Yet a third fungal pathogen of animal skin is *Batrachochytrium dendrobatidis*, a chytrid fungus found on frog skin and associated with amphibian population declines and even species extinction events throughout the world [6]. This chytrid fungus is from a basal group of fungi, quite phylogenetically divergent from either dermatophytes or *Malassezia* [7]. A fourth fungal pathogen of animal skin is *Geomyces destructans*, an ascomycete associated with white-nose syndrome and mortality of bats [8]. All four groups of fungi have been subject to whole genome analysis ([4], [9], [10]; http://www.broadinstitute.org), and their comparisons may reveal convergent solutions to adapting to such a unique environment as animal skin.

## Are *Malassezia* Capable of Mating?

Maybe! So far no sexual cycle has been observed for any of the 14 species of *Malassezia*. But they are phylogenetically related to the *Ustilago* genus of plant fungal pathogens, and these organisms are stimulated to complete their sexual cycle during infection of their plant hosts [11]. In turn, it is the *U. maydis* filamentous dikaryon produced by mating that is capable of infecting the host plant—the yeast form is not infectious. By analogy, the *Malassezia* species may complete their sexual cycle during growth on human skin. There is a precedent among fungi: skin was found to stimulate mating of *Candida albicans* [12]. As with *U. maydis*, mating may result in the production of novel *Malassezia* growth forms, such as hyphae, or differences in their secreted antigen repertoire. Based on whole genome analysis, a region corresponding to the mating type locus (*MAT*) has been identified for *M. globosa*, a species associated with dandruff [4]. One region encodes homeodomain transcription factors and the other a candidate pheromone and pheromone receptor, similar to other basidiomycete fungi, such as *U. maydis*. But interestingly, these two regions appear to be physically linked in *M. globosa*, which is more similar to the organization of the *MAT* locus of a related plant pathogen *Ustilago hordei* [13]. This suggests that if there is an extant sexual cycle for *M. globosa* that it is more likely to be bipolar with just two mating types, rather than tetrapolar with many mating types. Transitions from tetrapolar to bipolar mating configurations are common in the basidiomycetes, and may be the consequence of transitions from outbreeding to inbreeding as species specialize to a particular host niche [14]–[16]. The *M. globosa* genome also reveals other genes associated with sexual reproduction, such as those encoding key proteins required for meiosis [4]. Another indirect line of evidence that species in this genus may be sexual is the observation that certain lineages of *Malassezia furfur* appear to be hybrids, based on amplified fragment length polymorphism molecular analysis that reveals their genomes are a composite of two parental lineages [17]. These hybrids may have been produced by mating of isolates of opposite mating type. Next steps in the ongoing analysis of sexual potential will involve 1) population genetic tests for recombination as an indirect measure of sex, 2) direct tests of mating under laboratory conditions, 3) analysis of whether mating genes are expressed during fungal culture on skin, possibly leading to fungal sex occurring on our skin, resulting in virulence, and 4) characterization of the organization and allele diversity of the mating type locus.

## How Does *Malassezia* Interact with the Host?

Unlike its phylogenetically close relative, *U. maydis* (the causative agent of corn smut), *M. globosa* has a paucity of glycosyl hydrolases, suggesting it lacks the carbohydrate-degrading capacity found in plant pathogens. In contrast, *M. globosa* and a phylogenetically distant relative, the ascomycete human pathogen *C. albicans*, have a similar set of multicopy genes encoding secreted enzymes, including lipases and acid sphingomyelinases [4], [18]. *C. albicans* can survive in several body sites, including the skin where *M. globosa* is found. This set of secreted enzymes may enable these fungi to survive and even thrive on human skin. Within the *M. globosa* genome, extracellular lipases, acid sphingomyelinases, aspartyl proteases, and phospholipases are encoded by clusters of similar genes, suggesting recent gene duplication [19]. While some of these enzyme families are known to be involved in fungal pathogenesis, development of transformation and homologous recombination approaches will be necessary to test the roles of these enzymes. Are there any beneficial effects for the host to harbor these yeasts on the skin? This isn't known, but many individuals have used anti-fungal treatment for decades or longer without problems. If the fungi do confer benefits, they are either modest or at sites other than the scalp.

Since *Malassezia* species belong to the skin commensal flora, the host immune system will be regularly exposed to the fungi. IgG and IgM specific to *Malassezia* can be detected in healthy individuals [1]. The defective skin barrier in AE patients, both in lesional and non-lesional skin, fails to provide sufficient protection against microbes and allergens, facilitating interactions with *Malassezia* and the host immune system. Approximately 50% of adult patients with AE are sensitized to *Malassezia* reflected as allergen-specific IgE and T cell reactivity and/or positive atopy patch test reactions to the yeast [20]. Sensitization to *Malassezia* is most likely a combination of a dysfunctional skin barrier, genetic background, and environmental factors [21]. In addition, patients with AE lack appropriate induction of anti-microbial peptides, such as LL-37 and human beta-defensin-2 (HBD-2), which are produced in the skin as a first line of defense against bacteria, fungi, and some viruses, also suggesting an explanation for the frequent infections with *Staphylococcus aureus* in the skin of AE patients [22]. Moreover, the host interaction with *Malassezia* can stimulate the production of allergens. The release of *Malassezia sympodialis* allergens is indeed significantly higher at pH 6, reflecting the higher pH of skin from patients with AE compared to allergen release at pH 5.5, which is the normal skin pH [23].

Thirteen allergens from *Malassezia* species are reported to date by the official allergen nomenclature list (http://www.allergen.org). Interestingly, four of the *M. sympodialis* allergens, Mala s 1 and Mala s 7–9, encode proteins of unknown function without sequence homology to known allergens or to other known proteins [20]. Mala s 6, a cyclophilin, and Mala s 13, a thioredoxin, belong to a class of phylogenetically highly conserved proteins and are members of so called pan-allergen families. These proteins, together with Mala s 11 (a manganese-dependent superoxide dismutase), share a high degree of sequence identity to the corresponding human enzymes and might play an essential role in perpetuating skin inflammation of AE due to cross-reactivity [20], [24].

Human monocyte-derived DCs (MDDCs), as representatives of antigen presenting cells, can efficiently bind and rapidly internalize *M. sympodialis* as well as allergenic components from the yeast. This process is associated with maturation of the MDDCs, induction of lymphocyte proliferation, and of a Th2-like immune response. DCs can interact with natural killer (NK) cells in the skin, and *M. sympodialis* stimulates this interaction in patients with AE [25]. Furthermore, *M. sympodialis* enhances NK cell-induced DC maturation in healthy individuals [26]. NK and/or NKT cells might selectively eliminate DCs that have phagocytosed *Malassezia* before they activate the immune system, a function that might be impaired in AE.

The dominating symptom in AE is severe itching, which provokes scratching and increased inflammation. Mast cells most likely play a central role in this vicious cycle. Fungal products like zymosan can activate mast cells through TLR2, and cross-linking of the high-affinity IgE receptor, FcεRI, on mast cells leads to the release of potent inflammatory mediators, such as histamine, proteases, chemotactic factors, cytokines, and arachidonic acid metabolites. Recent data show that *M. sympodialis* can activate mast cells to release cysteinyl leukotrienes, enhance the mast cell IgE response, modulate MAPK activation, and by signaling through the TLR-2/MyD88 pathway alter IL-6 production [27]. Thus, these effects of *M. sympodialis* on mast cells most likely contribute to the inflammation and itching in AE.

A newly discovered route via which fungi interact with the host is the release of nanovesicles. *M. sympodialis* can release nanosized exosome-like extracellular vesicles with the capacity to induce inflammatory cytokine responses in both healthy individuals and patients with AE [28]. Theses vesicles can also carry allergen components from the yeast and induce significantly higher IL-4 production in patients with AE compared to healthy controls, suggesting that extracellular vesicles from *M. sympodialis* participate in host–microbe interactions in the pathogenesis of AE.

Many anti-dandruff shampoos contain anti-fungal agents as the active ingredients, and these agents remove the scalp-associated fungi as the scalp flaking symptoms improve. The root causes of dandruff may be similar to that of AE. Individuals with dandruff may have a skin barrier defect, as shown by their increased susceptibility to an externally applied irritating fatty acid, oleic acid [29]. *Malassezia* may have multiple roles in aggravating a barrier defect, including the production of increased amounts of irritating fatty acids as a result of lipase-mediated hydrolysis of sebum triglycerides [29].

There are likely differences in the ways in which dermatophytes and *Malassezia* interact with their environment. For example, the dermatophyte *Arthroderma benhamiae* has 26 genes encoding polyketide synthases and non-ribosomal peptide synthetases [9], whereas *M. globosa* has one copy of each gene. *A. benhamiae* encodes a hydrophobin gene that may affect interaction with the host [9]. We have not found a hydrophin gene within the *M. globosa* genome.

There are few animal models to explore the interaction of *Malassezia* with skin, although one could study natural infections of several non-human animals [5].

## How Do People Treat or Eradicate *Malassezia* from Their Skin?

The anti-fungal mechanism of action has recently been described for one commonly used anti-dandruff shampoo active ingredient, zinc pyrithione (ZPT). Based on the ionophore properties of pyrithione and the demonstrated increase in mammalian cell zinc levels upon ZPT treatment [30], it was expected that ZPT would act by delivering high intracellular zinc levels to inhibit fungal growth. With the use of *Saccharomyces cerevisiae* as a model yeast, ZPT was discovered instead to increase cellular copper levels, and genetics was used to demonstrate the biological activity of the elevated copper [31]. As is the case with copper-mediated growth inhibition in bacteria [32], [33], iron-sulfur clusters are the targets of ZPT. The role of copper in ZPT-mediated growth inhibition was also found with *M. globosa*, but the iron-sulfur theme was not tested due to experimental challenges with *Malassezia*. If these principles apply to the scalp, then the zinc from ZPT must be replaced by a scalp source of copper, either from the natural disintegration of skin cells or immune cells, which have been recently shown to exploit copper to control microbes [34].

Restoring the epidermal-barrier function and avoiding IgE sensitization are major targets for the prevention and treatment of AE [1]. It is important to stress that while there is some evidence that anti-fungal therapies may be of clinical benefit in patients with eczema associated with *M. sympodialis*, the vast majority of patients are treated with topical steroids or other immunosuppressive agents. For example, topical calcineurin inhibitors, including tacrolimus (Protopic) and pimecrolimus (Elidel), are commonly used to treat eczema based on their immunosuppressive properties. But these agents also inhibit fungal calcineurin, and exhibit anti-fungal activity that has been well documented for *Malassezia* species [35]. Thus, the efficacy of these topical agents may stem from a dual action to suppress host immune response and concomitantly inhibit growth of the fungus provoking host immune responses. Well-designed clinical trials are required to test this hypothesis. An alternative future treatment could be the use of selected cell-penetrating peptides, harmless toward mammalian cells but with anti-fungal activity [36].

## Conclusion

The study of host–pathogen interactions is more straightforward with skin pathogens than with systemic pathogens, as the pathogen is more easily studied in vivo. *Malassezia* are a ubiquitous component of the human skin microbiome and are associated with a myriad of skin problems, including dandruff in billions of people [37]. *Malassezia* are rarely found in places other than on animal skin, where they are such a common constituent of the flora that many (or possibly even all) warm-blooded animals harbor *Malassezia* on their skin. With the use of modern genomic and systems biology tools, we are poised to gain new insights in the interaction between humans and those eukaryotes with which we are most intimately associated, leading to perspectives on the duality of our symbiotic and antagonistic relationship.
